# SILAC-based quantitative proteomics using mass spectrometry quantifies endoplasmic reticulum stress in whole HeLa cells

**DOI:** 10.1242/dmm.040741

**Published:** 2019-11-11

**Authors:** Daniel N. Itzhak, Francesca Sacco, Nagarjuna Nagaraj, Stefka Tyanova, Matthias Mann, Marta Murgia

**Affiliations:** 1 Department of Proteomics and Signal Transduction, Max-Planck-Institute of Biochemistry, 82152 Martinsried, Germany; 2 Department of Proteomics, The Novo Nordisk Foundation Center for Protein Research, Faculty of Health Sciences, University of Copenhagen, DK-2100 Copenhagen, Denmark; 3 Department of Biomedical Sciences, University of Padova, 35121 Padua, Italy

**Keywords:** Proteomics, SILAC, Unfolded protein response, Endoplasmic reticulum stress, Tunicamycin

## Abstract

The unfolded protein response (UPR) involves extensive proteome remodeling in many cellular compartments. To date, a comprehensive analysis of the UPR has not been possible because of technological limitations. Here, we employ stable isotope labeling with amino acids in cell culture (SILAC)-based proteomics to quantify the response of over 6200 proteins to increasing concentrations of tunicamycin in HeLa cells. We further compare the effects of tunicamycin (5 µg/ml) to those of thapsigargin (1 µM) and DTT (2 mM), both activating the UPR through different mechanisms. This systematic quantification of the proteome-wide expression changes that follow proteostatic stress is a resource for the scientific community, enabling the discovery of novel players involved in the pathophysiology of the broad range of disorders linked to proteostasis. We identified increased expression in 38 proteins not previously linked to the UPR, of which 15 likely remediate ER stress, and the remainder may contribute to pathological outcomes. Unexpectedly, there are few strongly downregulated proteins, despite expression of the pro-apoptotic transcription factor CHOP, suggesting that IRE1-dependent mRNA decay (RIDD) has a limited contribution to ER stress-mediated cell death in our system.

## Abstract (precis)

**Summary:** A novel observation point of a familiar scenario: proteomic quantification of over 6200 proteins as a resource to further explore endoplasmic reticulum stress.

## INTRODUCTION

Endoplasmic reticulum (ER) stress is an impairment of cellular proteostasis, occurring when the cargo capacity of the ER is oversaturated as an effect of either increased functional demand or defective protein processing. An unfolded protein response (UPR) then ensues to restore protein homeostasis (Han and Kaufman, 2017; Walter and Ron, 2011).

The accumulation of misfolded proteins in the ER causes the activation of three transmembrane ER stress sensors, IRE1 (also known as ERN1), PERK and ATF6. For PERK and IRE1, activation is triggered by a reduction in the free concentration of the chaperone HSPA5 (also known as BiP), a negative regulator of ER stress sensor activation (Bertolotti et al., 2000; Kohno et al., 1993; Kozutsumi et al., 1988). Dissociation of HSPA5 enables homodimerization of both IRE1 and PERK, allowing them to autophosphorylate (Bertolotti et al., 2000). PERK then phosphorylates the eukaryotic translation initiation factor 2a (EIF2A), leading to EIF2A inactivation and repression of global protein synthesis (Harding et al., 1999). EIF2A phosphorylation also allows a selective increase in translation of certain mRNAs, including transcription factors ATF4 and CHOP (also known as DDIT3) (Harding et al., 2000). Autophosphorylation of IRE1 activates its endoribonuclease activity, which cleaves the mRNA of unspliced XBP1 (Itzhak et al., 2014). Ligation of the XBP1 exons by RTCB leads to the production of spliced XBP1, encoding a transcription factor that drives the expression of a coordinated set of UPR genes involved in protein folding and degradation (Back et al., 2005; Lu et al., 2014; Yoshida et al., 2001). The endoribonuclease has also been proposed to cleave other mRNAs, in a process termed regulated IRE1-dependent decay (RIDD), but the role of RIDD in mammalian cells is unclear (Bright et al., 2015; Han et al., 2009).

Upon sensing ER stress, the third ER stress sensor, ATF6, translocates to the Golgi (Okada et al., 2003; Schindler and Schekman, 2009), whereupon it is cleaved to release the N-terminal fragment that enters the nucleus to alter transcription (Haze et al., 1999; Wu et al., 2007). The combined action of these three branches of the UPR has been the subject of several studies, with different interpretations of the cellular output. These studies have focused on RNA expression levels (Acosta-Alvear et al., 2007; Bommiasamy et al., 2009; Sriburi et al., 2004), which is certain to be confounded by the effects of PERK action, whereas looking directly at protein levels would provide greater clarity on this matter. While initial proteomic studies were limited by the status of mass spectrometry (MS) technology (Bull and Thiede, 2012; Mintz et al., 2008), a more recent proteomic study used a recombinant system to uncover the direct targets of XBP1 and ATF6, purposefully excluding the effects of ER stress, IRE1 and PERK activation, as wells as ATF4 and CHOP expression (Shoulders et al., 2013). MS-based proteomics was also applied to tunicamycin treatment, albeit combined with chemical proteomics and with a specific focus on the ER (Fujisawa et al., 2018).

The global cellular reprogramming that accompanies the imbalance of proteostasis is complex and partially heterogeneous in different cells (Saito and Imaizumi, 2018a). Several features of ER stress play a common role in the cell pathology of profoundly different diseases, such as type 1 and type 2 diabetes, and cancer, particularly cancers of a secretory cell origin (Harding et al., 2001; Marre et al., 2018; Yadav et al., 2014). However, despite an increasing number of proteins and signaling events being implicated in these connections, many mechanistic links to disease and therapy are still missing.

We employ MS-based shotgun proteomics to measure changes in protein abundance at the whole-cell level during ER stress, using high-accuracy quantitation afforded by stable isotope labeling by amino acids in cell culture (SILAC) (Ong et al., 2002). We initially analyze the effects of treatments that induce proteostatic stress through entirely different mechanisms, namely the N-glycosylation-inhibiting drug tunicamycin, the reducing agent dithiothreitol (DTT) and the sarco/endoplasmic reticulum Ca^2+^-ATPase (SERCA) pump inhibitor thapsigargin. We then analyze the proteomic remodeling induced by treatment with increasing concentrations of tunicamycin in HeLa, a well-characterized and widely used model where all three main pathways controlling the UPR are functioning and highly responsive. Our strategy targets the whole-cell proteome, not selected organellar fractions, to offer a broad view of the consequences of stress in different cell compartments. We have quantified over 6200 proteins and 90,000 peptides using SILAC-based proteomics. By providing a system view of the UPR, we generate a resource to determine and investigate the role of ER stress in a broad range of pathophysiological contexts.

## RESULTS

### Proteomic workflow for the generation of a SILAC-based resource dataset of the unfolded protein response

**Fig. 1.**
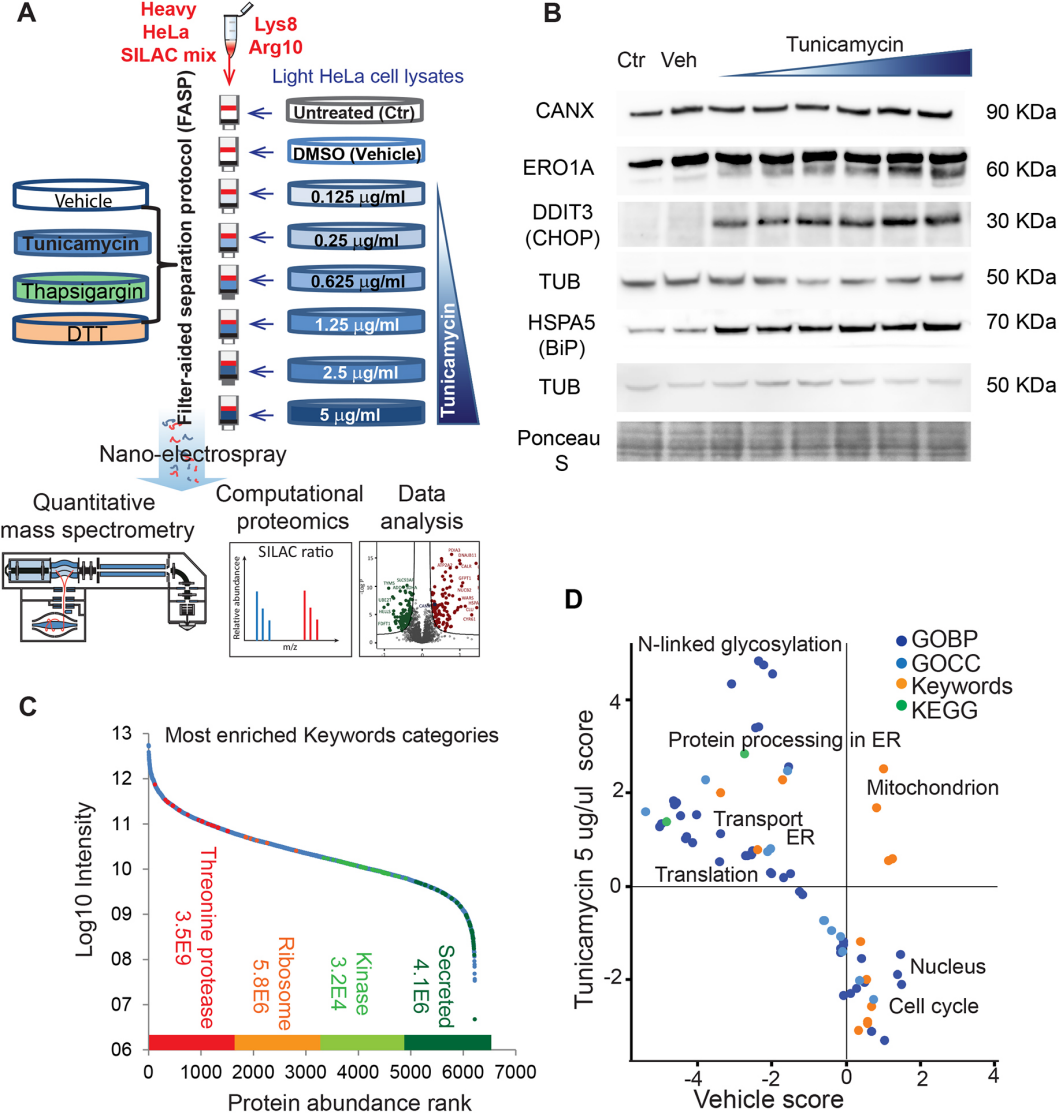
**SILAC-based quantitative proteomics workflow.** (A) Experimental design for the analyses of untreated (Ctr), DMSO-treated (Vehicle, Veh) and stressor-treated samples. HeLa cells grown in medium containing natural (light) amino acids (represented to the right) were treated with increasing concentrations of tunicamycin (in representative shades of blue). To compare the effects of different stressors, cells were also treated with thapsigargin (represented in green) and DTT (orange). A mixture of tunicamycin-treated and untreated HeLa cells labeled via SILAC with heavy lysine and arginine served as an internal standard (represented in red, see text and Materials and Methods section). The light lysates and heavy standards were mixed in a 1:1 ratio, processed by FASP and digested with trypsin. The resulting peptides were analyzed by high-resolution MS and raw files processed using the MaxQuant environment. (B) Western blot analysis with antibodies specific for known ER stress markers of the lysates used for MS analysis. Biological replicate 1, probed with antibodies specific for calnexin (CANX), ERO1-like protein alpha (ERO1A) and DNA damage-inducible transcript 3 protein (DDIT3, CHOP) and biological replicate 2, probed with anti-HSPA5 (BiP), are shown. Two different blots are shown with respective loading controls. (C) Dynamic range of the quantified proteome. The light intensity (L, see Table S1) of all proteins quantified by SILAC ratio is shown. The enriched categories with highest score (determined by Fisher's exact test using false discovery rate, FDR, at 0.04) and their respective *P*-value are shown for proteins of high (quartile 1, red), medium (quartiles 2 and 3, orange and light green) and low intensity (quartile 4, dark green). (D) Scatter plot of 2D annotation enrichment showing differences between the proteomes of DMSO- and tunicamycin (5 μg/ml)-treated cells. The experiments were performed in three independent biological replicates and each data point was analyzed in technical triplicates. The calculation of significance is described in the Materials and Methods section. Gene Ontology Biological Process (GOBP), Gene Ontology Cellular Component (GOCC), UniProt Keywords and Kyoto Encyclopedia of Genes and Genomes (KEGG) annotations were analyzed (see legend).

To achieve a high-accuracy analysis of proteome remodeling in response to ER stress, we used a quantitative proteomics approach based on the SILAC technology (Fig. 1A; Table S1, complete dataset with all biological and technical replicates). We impaired protein N-glycosylation in HeLa cells using increasing amounts of the nucleoside-type antibiotic tunicamycin, which causes the accumulation of incompletely processed glycoproteins in the ER and the activation of the UPR (Duksin and Mahoney, 1982; Kozutsumi et al., 1988). To dissect the features of low- and high-level UPR activation, we used a broad drug concentration range, from 0.125 μg/ml to 5 μg/ml. HeLa cells cultured in medium containing light unlabeled lysine and arginine (represented to the right in Fig. 1) were left untreated (Ctr), or treated with either DMSO (the vehicle, Veh, in which tunicamycin is resuspended) or increasing concentrations of tunicamycin for 18 h (Saito et al., 2009), followed by lysis in an SDS-containing buffer. We also compared the effects of tunicamycin to those of other stressors activating the UPR, namely thapsigargin and DTT. The heavy SILAC standard (represented in red) serves as a fixed reference to detect changes occurring in the light HeLa cells in a ratio-based fashion. Therefore, it should ideally contain all possible cellular proteins expressed in HeLa cells, in order to provide the heavy-labeled counterpart of each peptide and thus allowing measurement of SILAC ratios. To this aim we prepared the SILAC standard by mixing lysates of heavy HeLa cells treated with tunicamycin and other stressors that induce different sets of proteins, such as DTT and thapsigargin, as well as untreated and vehicle-treated cells. For MS analysis, 100 μg of each lysate of light tunicamycin-treated HeLa were mixed with the same amount of heavy standard and processed using the filter-aided sample preparation (FASP) method (Wiśniewski et al., 2009). We verified the effects of tunicamycin by analyzing the expression of the known ER stress markers calnexin (CANX), a calcium-binding ER chaperone, ERO1-like protein alpha (ERO1A), an ER oxidoreductase involved in disulfide bond formation, the ER chaperone HSPA5 (BiP) and the DNA damage-inducible transcript 3 protein (DDIT3, CHOP) the same lysates by western blotting (Fig. 1B) (Walter and Ron, 2011). Gel band densitometry was used to quantify the changes in expression of the proteins shown in the western blot (Fig. S1).

The purified peptides were analyzed by reverse-phase liquid chromatography (LC) coupled through a nano-electrospray source to a quadrupole-Orbitrap mass spectrometer. We performed the experiment in three independent biological replicates; in the case of tunicamycin treatment, each sample was further analyzed in technical triplicates. We used the MaxQuant software environment for the quantification of SILAC pairs corresponding to the light (experiment) and heavy (SILAC standard) tryptic peptides (Tyanova et al., 2014, 2016). Using the ‘Match between runs’ feature, based on retention time alignment, we transferred feature identifications among samples, thus increasing the number of identified peptides per sample. This led to the identification of 10,016 proteins and to the accurate quantification of 6210 of them through the SILAC ratios (as both the light and heavy version of each protein needs to be quantified at high confidence in order to yield a valid ratio), encompassing categories of highly abundant ribosomal proteins and proteasome subunits and low copy secreted proteins (Fig. 1C). Transmembrane proteins accounted for 15% of all quantified proteins on average (UniProt Keyword annotations) independent of treatment. In vehicle-treated cells we quantified 736 transmembrane proteins (median SILAC ratio 1.02±0.24, median±s.d.). At the highest tunicamycin dose, we quantified 749 transmembrane proteins (median SILAC ratio 1.03±0.26). Modifications occurring on residues other than lysine and arginine (trypsin cleavage sites), such as glycosylation, are not known to directly affect trypsin digestion. It should also be noted that that only a small fraction of the proteome is estimated to be N-glycosylated and glycoprotein-targeting proteomics is based on specific enrichment methods (Zielinska et al., 2010). To verify the effects of UPR activation at the whole proteome level, we employed 2D annotation enrichment, an algorithm that calculates enrichments in two datasets compared to a background proteome, in this case the human proteome (Cox and Mann, 2012). This approach enabled the identification of Gene Ontology (GO) terms, Kyoto Encyclopedia of Genes and Genomes (KEGG) pathways and Keywords significantly enriched in a comparison between control and stressed cells. Protein annotations such as N-linked glycosylation and protein processing in ER were over-represented in the proteome of stressed cells. Conversely, vehicle-treated cells were specifically enriched in proteins annotated to the cell cycle, confirming that control cells, unlike stressed cells, were actively proliferating (Fig. 1D; Table S2, significantly enriched terms from the 2D annotation enrichment). The protein list of all three biological replicates, showing the median of three technical replicates per data point, is available as a resource in Table S3 (complete dataset averaged by biological replicate). This dataset format can be easily imported into the Perseus environment of MaxQuant (Tyanova et al., 2016) and browsed using the profile plot option.

### Proteomic comparison of the effects of different agents causing ER stress

**Fig. 2.**
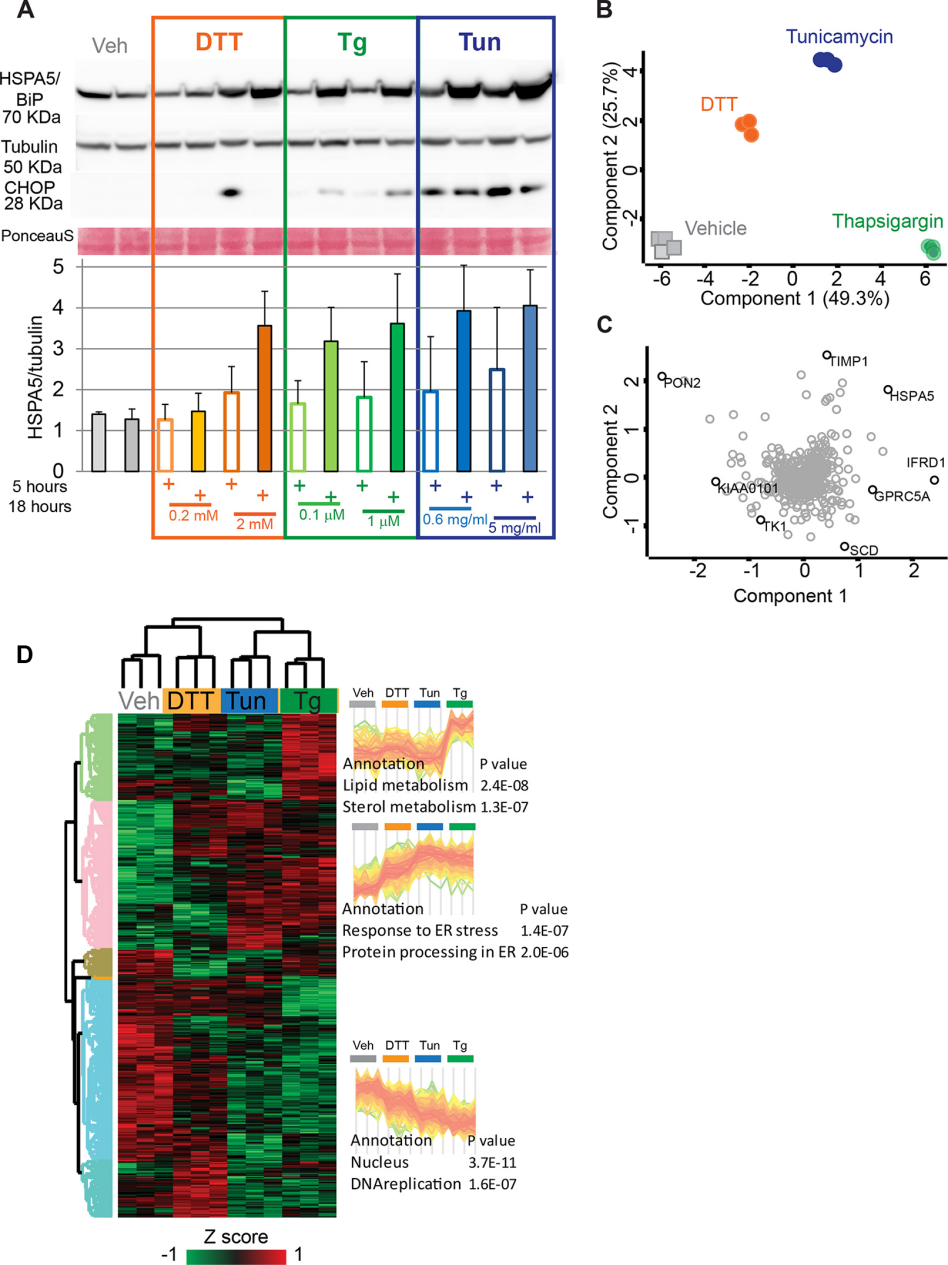
**Effects of different stressors on UPR activation at the** **proteome level.** (A) Time course and dose-dependence of different stressors. HeLa cells were treated for 5 and 18 h as indicated with low (DTT 0.2 µM, thapsigargin 0.1 µm, tunicamycin 0.625 µg/ml) and high dose (DTT 2 mM, thapsigargin 1 µm, tunicamycin 5 µg/ml) of stressors. Western blot analysis shows the expression of CHOP (DDIT3) and HSPA5. Tubulin and Ponceau S are shown as loading controls. The experiment was carried out in three replicates. The HSPA5:tubulin ratio (*N*=3, mean±s.d.), quantified using ImageJ, is shown as a bar graph. Controls at 18 h are shown. (B) Principal component analysis (PCA) comparing the proteome remodeling induced by tunicamycin (5 μg/ml), DTT (2 mM) and thapsigargin (1 µM). Vehicle-treated cells were used as controls. Each treatment was performed in biological triplicates. Data were filtered for 100% valid values in ANOVA-significant proteins (543 proteins). (C) Loadings of B, showing the major separators into components one and two (*x*- and *y*-axis, respectively). TIMP1, metalloproteinase inhibitor 1; HSPA5, endoplasmic reticulum chaperone BiP; IFRD1, interferon-related developmental regulator 1; GPRC5A, retinoic acid-induced protein 3; SCD, stearoyl-CoA desaturase 5; PON2, serum paraoxonase/arylesterase 2; KIAA0101, PCNA-associated factor (also known as PCLAF); TK1, thymidine kinase. (D) Unsupervised clustering of ANOVA-significant proteins showing three main clusters, with profiles and corresponding relative enrichments, indicated to the right. Enrichment is based on Fisher's exact test of keyword annotations at 0.04 FDR.

Perturbations of homeostatic conditions in the ER activate the stress sensors IRE1, PERK and ATF6, which trigger the UPR. Different chemical stressors and defective protein folding in the lumen of the ER, due to mutation or overload, elicit ER stress with distinct features. Drugs with different mode of action also activate parallel pathways unrelated to the UPR (Bergmann et al., 2018). We thus initially set out to analyze the effects of three chemically unrelated stressors, namely tunicamycin, DTT and thapsigargin, that activate the UPR through different mechanisms. Tunicamycin inhibits N-linked glycosylation and DTT reduces disulfide bonds in the whole cell, thereby also preventing proper folding of newly synthesized proteins and causing ER stress. Thapsigargin inhibits the SERCA ATPase, thus emptying the ER calcium stores and causing a profound imbalance in calcium homeostasis with major effects on protein trafficking. We tested different doses of these stressors and analyzed their effect on the induction of the UPR. As these compounds have different modes of action and cause ER stress with different kinetics, we tested two time points after initiation of treatment, 5 h and 18 h. As readout for the ability of the drugs to trigger a full-blown UPR, we used the expression of the key ER stress sensor HSPA5 and of the transcription factor CHOP. Based on normalized HSPA5 expression, maximal UPR activation was reached after 18 h of treatment for all three stressors. CHOP expression was only evident at 18 h for thapsigargin, whereas it was evident only at 5 h for DTT and maximal at 5 h for tunicamycin, though still present at 18 h of treatment. These observations confirm that DTT has a faster mode of action compared to the other two drugs (Fig. 2A). These observations are in line with previous reports describing the UPR in HeLa cells, with minor differences (i.e. the late effect of thapsigargin on CHOP expression in our dataset) that might be due to the clonal heterogeneity of this cell line. At the concentration range we used, all three stressors have been previously shown to consistently activate HSPA5, as well as the HSP90B1 (also known as GRP94) chaperone, without causing detectable cell death at 24 h of treatment (Shinjo et al., 2013). We thus set out to analyze the proteome of HeLa cells treated for 18 h with 2 mM DTT, 1 µM thapsigargin and 5 µg/ml tunicamycin. Under these conditions, HeLa cell samples treated with the different drugs were clearly separated by principal component analysis (PCA) and diverged from untreated controls along component one (*x*-axis, Fig. 2B). Furthermore, cells treated with each stressor grouped together and, in particular, those treated with thapsigargin were separated from those treated with tunicamycin and DTT along component two (*y*-axis, Fig. 2B). The proteins driving most of the separation among the proteomes of cells with different treatments in the PCA can be visualized as loadings. Among the main loadings of component one was the ER chaperone HSPA5, confirming that the UPR had been activated, as well as cell cycle and growth regulators. The expression of the acyl-CoA desaturase SCD and of the metalloproteinase inhibitor TIMP1, by contrast, was a main driver of the separation between thapsigargin- and tunicamycin-treated cells, respectively (Fig. 2C).

To further investigate the proteome remodeling caused by different stressors, we performed ANOVA using a permutation-based false discovery rate of 0.05, comparing the SILAC ratios obtained upon treatment with different stressors. Unsupervised hierarchical clustering of all ANOVA significant proteins showed that vehicle-treated cells had the highest expression of the enriched DNA replication protein cluster (*P*<10^−6^), indicating that the stressors inhibit DNA replication (Fig. 2D, specific enrichments and cluster shapes on the right). This analysis highlights different effects of each stressor. Tunicamycin shows the strongest increase in proteins annotated to ER stress (annotation ‘Response to ER stress’ and ‘Protein processing in ER’) compared to the other stressors. Based on the large number of proteins showing this behavior in the cluster analysis, as well as on the results in Fig. 2A, we conclude that tunicamycin is a stronger inducer of the UPR than the other stressors under our experimental conditions. All treatments, DTT with a comparatively milder effect, cause a downregulation of proteins annotated to DNA replication. Interestingly, only thapsigargin causes a significant increase in the expression of proteins involved in lipid metabolism, which appears unrelated to UPR activation, as tunicamycin and DTT are indistinguishable from control cells in this case. The complete list of enriched annotations in all clusters of Fig. 2D is shown in Table S4.

### Effects of increasing doses of tunicamycin in HeLa cells

**Fig. 3.**
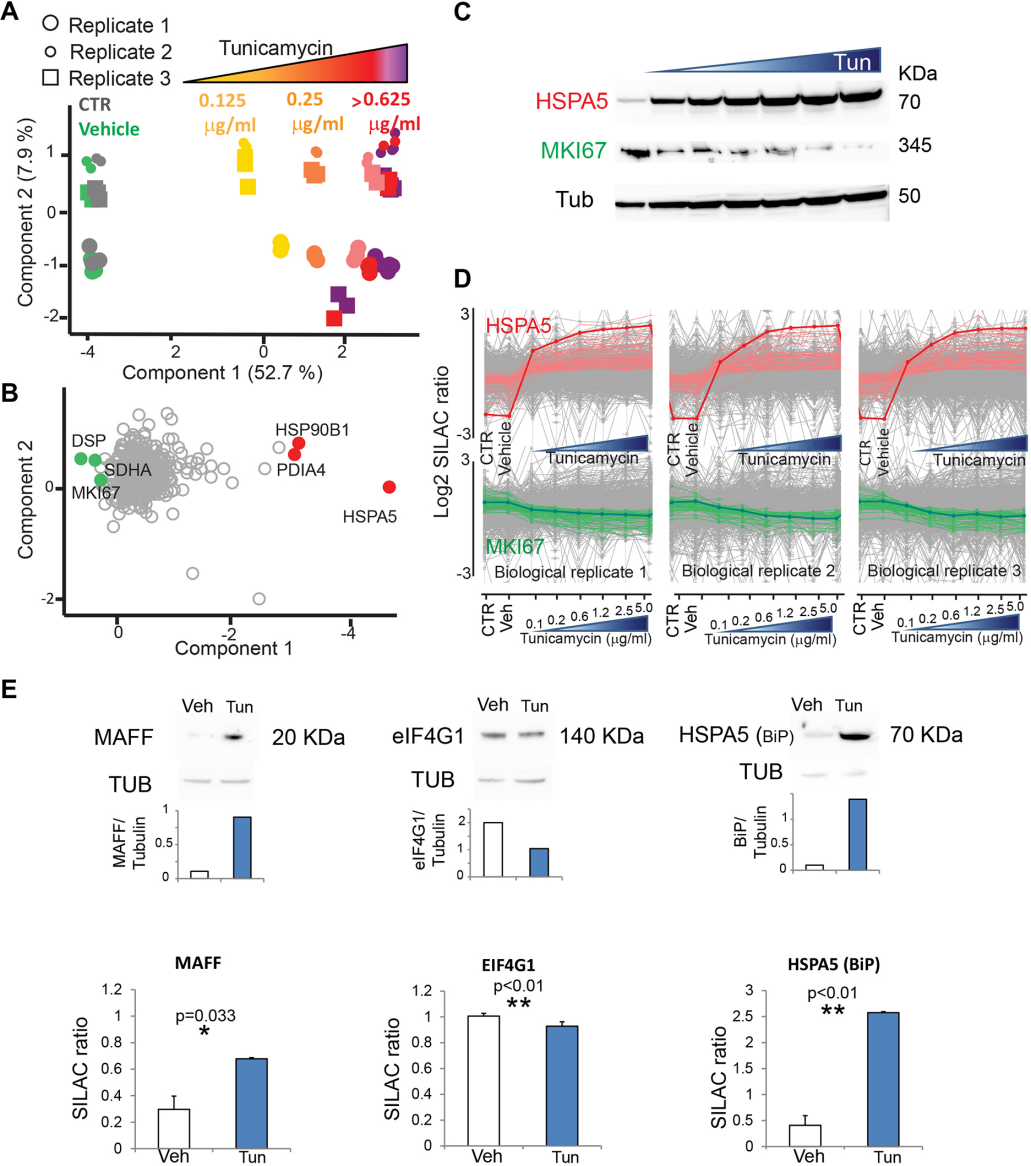
**Global proteomic view of the UPR upon treatment with increasing doses of tunicamycin.** (A) Principal component analysis (PCA) showing graded features of HeLa cell response to tunicamycin. The proteomes of tunicamycin-treated samples segregate in a concentration-dependent fashion from those of controls and vehicle-treated cells along component 1. Data were filtered for 100% valid values (600 proteins). Three independent biological replicates segregate along component 2. (B) Loadings of A showing proteins that strongly drive the segregation into PCA component 1. HSP90B1, endoplasmin; PDIA4, protein disulfide-isomerase A4; HSPA5; DSP, desmoplakin; SDHA, succinate dehydrogenase; MKI67, proliferation marker protein Ki-67. (C) Western blot showing the mirror-like expression of the two of the main driver proteins in panel B, HSPA5 and MKI67, in response to increasing concentration of tunicamycin. Tubulin expression is shown as loading control. (D) Most similar expression profiles to HSPA5 and MKI67 (thick red and green lines, respectively) among the proteins in the dataset. Profiles derived from the median of triplicates of three biological replicates are shown. The *x* axis shows the different treatments as indicated. (E) Comparison of the expression of selected proteins quantified by western blotting and corresponding densitometry (top) and by MS-based proteomics (bottom, *N*=3 biological replicates, mean±s.d. with Student's *t*-test; *P<0.05; **P<0.01). The MAFF blot was subjected to automatic contrast enhancement due to low signal.

After validating tunicamycin as having the strongest effect of the three chemical stressors tested on UPR activation under our experimental conditions, we focused on the effects of this stressor in HeLa cells. We used increasing doses of the drug ranging from 0.125 μg/ml to the maximal dose of 5 µg/ml, corresponding to that used in Fig. 2. PCA of all three biological and technical replicates showed a net separation of tunicamycin-treated samples from controls (Fig. 3A). Samples treated with tunicamycin distributed along component one, showing a dose-dependent separation of samples treated with up to 1.25 μg/ml. Samples treated with higher doses occupied a partially overlapping area, separated from those at low dose. Different replicates distributed along component 2, which accounts for less than 8% of the observed difference (Fig. 3B). The loadings of component one, which capture the differences between controls and tunicamycin-treated cells, include known cell-cycle proteins and UPR players, respectively. The strongest differentiator of tunicamycin-treated cells was HSPA5, confirming the ability of SILAC-based proteomics to recapitulate known properties of the UPR while extending the analysis to the global proteome level. In our dataset, we could quantify only two peptides of the known UPR effector XBP1 (VVVAAAPNPADGTP and GASPEAASGGLPQARK), both located in the N-terminal region. Thus, we could not specifically measure the stress-responsive spliced form (Yoshida, 2007). This might be due to low recovery and/or concentration in the lysate, and rapid degradation in our workflow. In addition, low ‘flyability’ of XBP1 peptides in the mass spectrometer, due to insufficient ionization, may contribute to the poor quantification of this specific protein. MKI67, a broadly used proliferation marker, was a major driver of the control and vehicle samples, a feature which is expected in actively cycling HeLa cells in the absence of stressors (see also Fig. 2D). We confirmed by western blotting the effect of tunicamycin on MKI67 expression (Fig. 3C). We then asked which proteins have a similar behavior to HSPA5 and MKI67 in response to various degrees of ER stress. We used the expression profile of these two drivers to search, via a correlation analysis, the proteins with the most similar expression profiles. Fig. 3D shows the most closely correlated expression profiles to HSPA5 and MKI67 in three biological replicates. The full list is shown in Table S5 (100 profiles most similar to HSPA5 by correlation) and Table S6 (100 profiles most similar to MKI67 by correlation).

The analysis of the UPR using SILAC-based proteomics provides a reliable and robust quantification of protein expression at high accuracy. In our dataset, 975 proteins out of 6220 undergo an increase in expression higher than 10% at tunicamycin doses >1.25 µg/ml. The expression of 1304 proteins decreases more than 10% under those conditions. Some of these proteins are expressed at very low level and could not be quantified in all replicates. We validated the expression changes of selected proteins using western blotting (Fig. 3E). We focused on MAFF, a basic region leucine zipper (bZIP)-type transcription factor not previously linked to the UPR, for which we measured an increase of SILAC ratio upon tunicamycin treatment. This finding was confirmed using western blotting. The translation initiation factor eIF4G showed a small but significant decrease in expression upon tunicamycin treatment, which had not been reported before. As predicted, HSPA5 showed a >fivefold upregulation of SILAC ratio under these conditions (Fig. 3E).

### Novel features of the UPR highlighted by SILAC-based proteomics

**Fig. 4.**
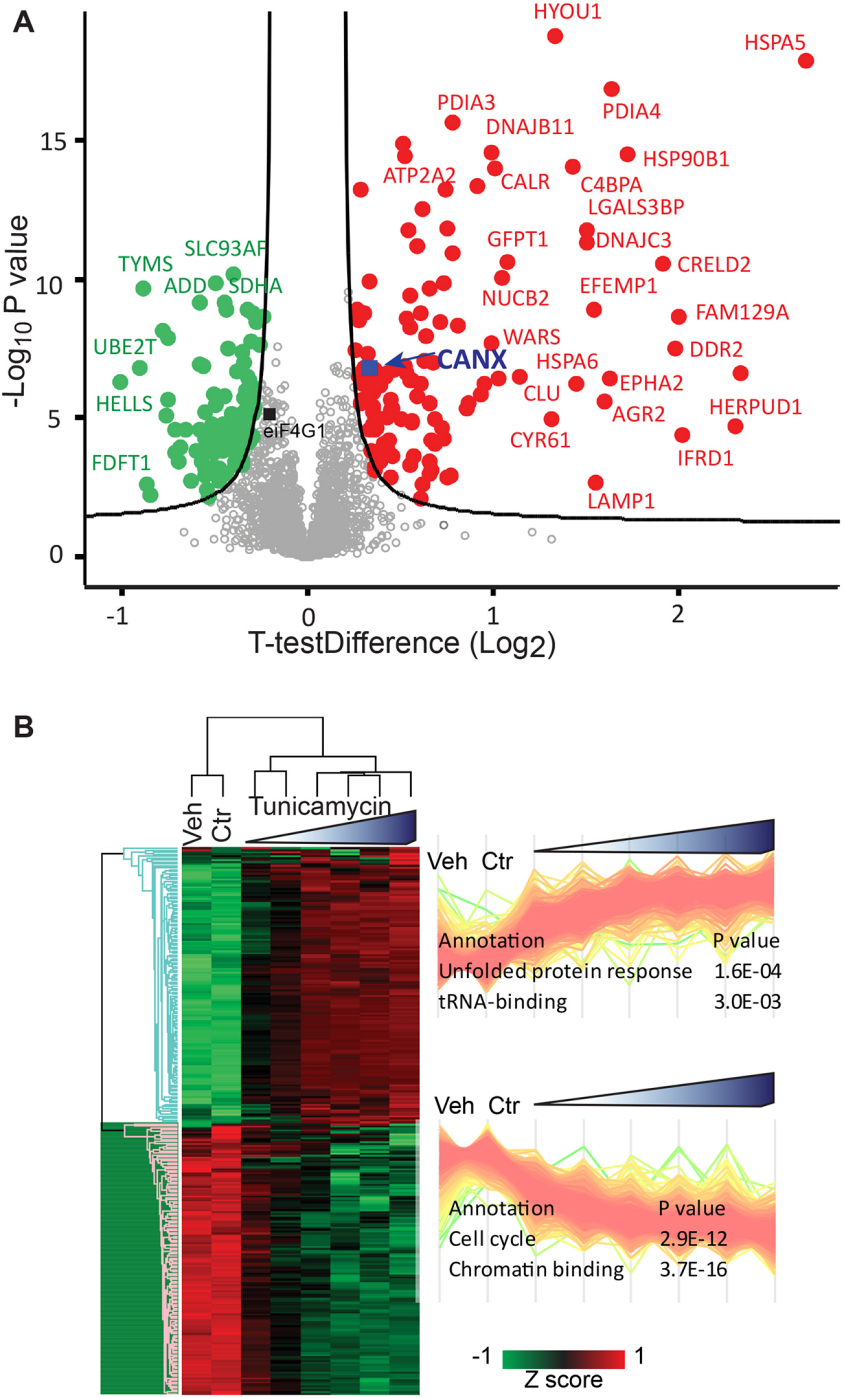
**Proteome remodeling upon tunicamycin treatment involves hundreds of significant expression changes.** (A) Volcano plot comparing protein expression levels in samples treated with vehicle to those with the highest tunicamycin concentration (5 μg/ml). Proteins significantly upregulated upon tunicamycin treatment are shown as red dots, those downregulated as green dots. The curve is derived at FDR=0.05 and *s*_0_=0.5 as described in the Materials and Methods section. Under these conditions, the UPR protein calnexin is significantly upregulated as expected (CANX, blue square, see text). The dataset was filtered for 50% valid values in at least one group. eIF4G1 is marked with a black square, MAFF and CHOP were excluded by the filtering procedure (see Fig. 3E). (B) Unsupervised hierarchical clustering of 238 proteins with significantly different levels of expression between DMSO- and tunicamycin (5 μg/ml)-treated HeLa cells (median of *N*=3 biological replicates). Profiles of the proteins in the clusters and corresponding relative enrichments are indicated to the right. Enrichment is based on Fisher's exact test of keyword annotations at FDR=0.04.

We subsequently used our resource dataset for the large-scale identification of proteins that have a significantly different expression in tunicamycin-treated and untreated cells. To this end, we performed a Student's *t*-test using permutation-based false discovery rate (FDR) at 0.05, using the respective SILAC ratios. Fig. 4A shows the volcano plot comparing vehicle-treated cells with those treated with 5 µg/ml tunicamycin, the highest dose showing the strongest effects on UPR activation. We performed the same analysis comparing cells treated with 0.625 µg/ml tunicamycin with vehicle-treated cells (Fig. S2A). ER chaperones and foldases, such as HSPA5 and various disulfide isomerase (PDI) family members were among the most significantly upregulated proteins at both concentrations, together with a subset of N-glycosylated membrane and secreted proteins (e.g. DDR2). We set the stringency of our cut-off values using previous knowledge of the UPR in addition to statistical criteria (Delom et al., 2007). We first confirmed that the ER chaperone calnexin undergoes a small but significant upregulation under our test conditions using immunoblot analysis (Fig. S2B,C). We then chose parameters to ensure that this known UPR target was close to the cutoff but retained within the significantly upregulated proteins. Therefore our significance cut-off allows us to discover potential novel UPR targets with high confidence. The resulting dataset greatly enlarges our view on the effects of tunicamycin in comparison to previous biological knowledge and proteomics datasets present in the literature (Fig. S2D) (Bull and Thiede, 2012; Mintz et al., 2008).

Altogether, we derived a list of 238 proteins that are significantly modulated by treatment with 5 µg/ml tunicamycin, shown as a heat map of the SILAC ratios after clustering (Fig. 4B). This procedure separates two clusters, showing that proteins significantly upregulated upon tunicamycin treatment are specifically enriched in ER and protein processing annotation, whereas nuclear and cell­ cycle annotations prevail among downregulated proteins. The corresponding clusters for cells treated with a concentration of 0.625 µg/ml tunicamycin are shown in Fig. S2E. The complete list of enriched terms is provided in Table S7 (annotation enrichments resulting from the cluster analysis in Fig. 3) and the proteins significantly up- and downregulated by tunicamycin in Table S8 (significantly upregulated proteins with complete annotations) and Table S9 (significantly downregulated proteins with complete annotations). In both tables, proteins that do not undergo statistically significant changes in expression at the concentration of 0.625 µg/ml tunicamycin are indicated. The profile plot graphs of proteins significantly up- and downregulated at 5 µg/ml tunicamycin are shown in Fig. S2F.

To categorize the list of upregulated proteins (listed and annotated in Table S8), the proteins were labeled as previously being identified as a UPR target gene, defined as any gene whose expression changes upon ER stress, or having been identified in a proteomic study of tunicamycin­-treated cells. This definition likely includes proteins whose expression is induced by secondary effects, as no comparison is made to cells without a functional UPR. Nonetheless, 70 of 133 proteins were known UPR targets (Table S8). Therefore, genes with related functions, as listed in the UniProt database, were assigned as novel UPR target genes if they also fit into one of these groups: ER import, folding (HSP40, HSP70 and HSP90, also known as DNAJB1, HSPA1A/B and HSP90AB1, respectively), glycosylation and lectin-assisted folding, disulfide redox, quality control and degradation, anterograde trafficking/Golgi (Shoulders et al., 2013; Sriburi et al., 2007). This identified 15 novel UPR targets, which include membrane transporters, transcription factors and signal transducers. Of the remaining proteins, four are involved in autophagy, a process known to be activated by treatment with tunicamycin, and proposed to be by direct activation by IRE1 (Ogata et al., 2006; Pattingre et al., 2009). Three upregulated proteins are annotated as pro-apoptotic, consistent with the observation that prolonged exposure to tunicamycin causes programmed cell death (Zinszner et al., 1998).

For the remaining genes, no obvious functional similarity to known UPR targets was apparent. However, the presence of a transmembrane domain, signal peptide, disulfide bonds or glycosylation would suggest passage through the secretory pathway and hence potential cargo proteins affected by tunicamycin treatment. 23 proteins met these criteria, of which 9 were annotated as plasma membrane proteins, 11 secreted, while one had secreted and plasma membrane isoforms. Two of these proteins had no subcellular location annotation beyond membrane. We were unable to categorize 18 proteins that were significantly upregulated (Table S8).

It has been shown that IRE1 can cleave mRNAs other than XBP1 during the UPR, which is suggested to reduce protein load on the ER (Hollien et al., 2009; Hollien and Weissman, 2006) and may contribute to apoptosis under irremediable ER stress (Han et al., 2009). These studies have focused on the mRNA levels of these genes, leaving the question open as to how effective RIDD is at reducing the protein load. Here, we looked at RIDD targets identified previously by microarray experiments, to assess if their protein levels were also reduced (Table S9). We annotated our dataset using previously identified RIDD targets (Bright et al., 2015). Of 186 RIDD targets that mapped to our dataset, 17 were in our list of 105 significantly downregulated genes, representing a statistically significant 2.3-fold enrichment in RIDD targets amongst downregulated genes (*P*=0.0006). Another 16 RIDD targets were in our list of 133 significantly upregulated genes, a 1.7-fold enrichment (*P*=0.0128). Overall, RIDD targets appear evenly distributed among the dataset (Fig. S3A). Taken together, these data highlight a poor correlation between protein and mRNA levels of previously identified RIDD targets. Our results thus suggest that degradation of RIDD targets at the mRNA level does not necessarily lead to a concomitant decrease at the protein level that persists 18 h after induction of ER stress. This is further supported by the low overall correlation of protein and mRNA levels of cells treated with tunicamycin (Fig. S3B). RIDD has been postulated to degrade mRNAs that make up the secretory pathway following commitment to cell death (Maurel et al., 2014). Hence, these mRNAs must encode proteins that pass through the secretory pathway or that are residents thereof. It is therefore possible that RIDD targets are cell type-specific, and would depend on the proteins passing through the secretory pathway. To assess this possibility we annotated the dataset with Gene Ontology terms and performed 1D annotation enrichment on the downregulated genes (Cox and Mann, 2012). However, the only enrichment among downregulated proteins was for the keyword glycoprotein (2.7-fold, *P*=0.027) and no secretion-related term was significant. Recent studies have suggested that RIDD is sequence-specific in mammalian cells (Bright et al., 2015; Moore and Hollien, 2015) and we therefore looked for XBP1-like stem loops in the significantly downregulated genes. We found 186 genes in our dataset containing a sequence that could be recognized by IRE1. Ten of these were downregulated but this enrichment was not statistically significant. Were IRE1 to exhibit non-specific cleavage of mRNAs to cause downregulation of a large number of proteins, this effect would not appear to be substantial, given there are only 105 significantly downregulated genes whose downregulation is minimal in comparison to the upregulation of canonical UPR genes (Fig. 4A). In summary, these data do not support a major role for RIDD in downregulation of proteins that might contribute to ER stress-mediated cell death. However, reduction of mRNAs encoding components of the secretory pathway to alleviate ER stress earlier in the UPR is unlikely to be seen in this data, especially if this effect is only short-lived.

### Interaction network of tunicamycin-regulated proteins

**Fig. 5.**
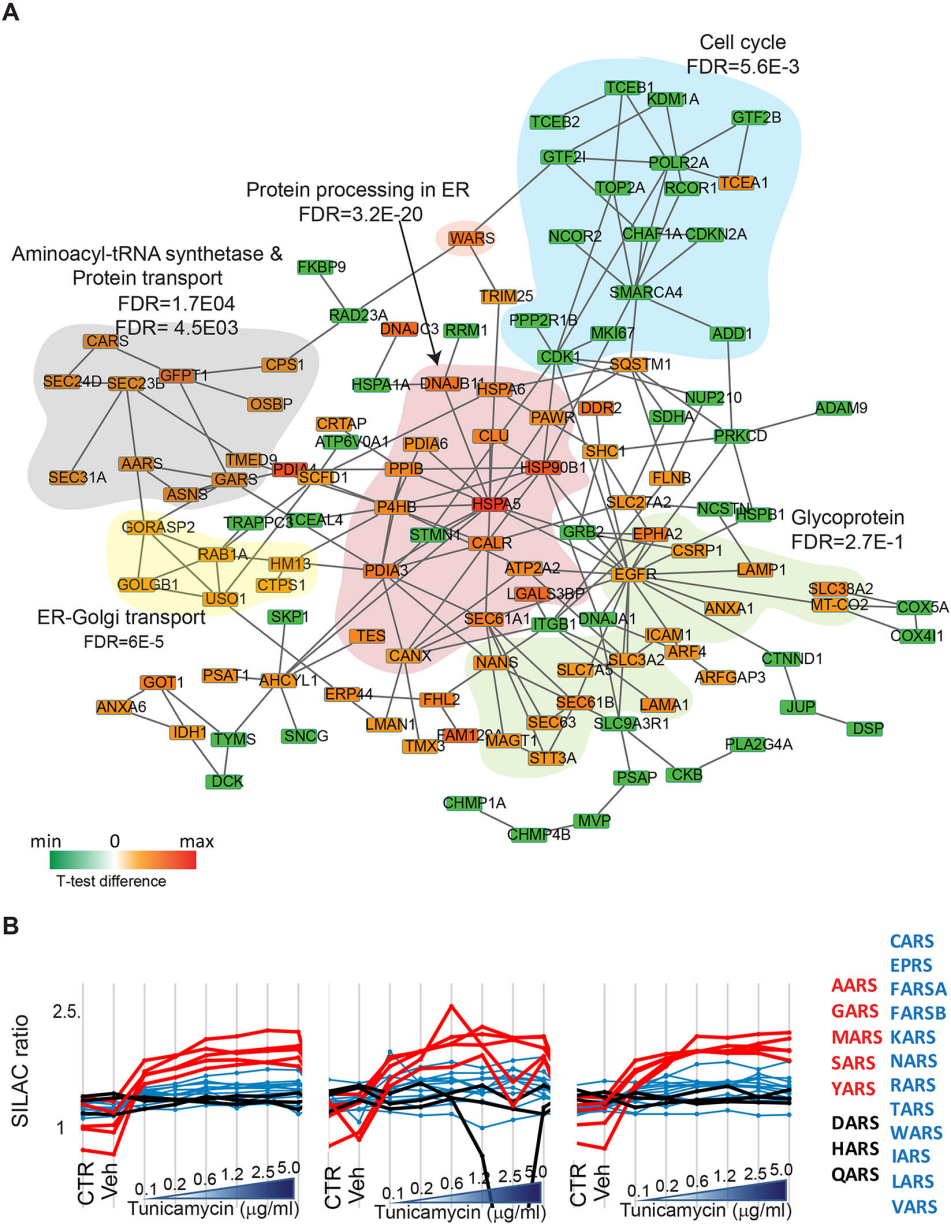
**Physical interaction network of proteins significantly regulated upon tunicamycin treatment.** (A) Upregulated proteins are represented in red, downregulated proteins in green according to the indicated color scale. Lines represent physical interaction. Functional subgroups are marked as colored areas. (B) Profile plots showing the coordinated expression changes of tRNA ligases upon tunicamycin-mediated UPR activation. The five tRNA ligases with the highest expression changes are indicated by red lines and font, other tRNA ligases showing increases in expression are shown in blue. tRNA ligases for which no increase in expression upon tunicamycin treatment was detected are indicated in black.

We next performed a physical interaction network analysis of tunicamycin-regulated proteins in the STRING database (Szklarczyk et al., 2015) (Fig. 5A; ‘known interactions experimentally determined’, see Materials and Methods). Our analysis highlights a large group of proteins with increasing expression upon tunicamycin treatment that are highly interconnected, consisting of ER chaperones and foldases involved in protein processing in the ER (Fig. 5A, red area). Interestingly, this cluster shows a number of interactions with another group of proteins that are upregulated during ER stress, namely those involved in aminoacyl tRNA biosynthesis (Fig. 5A, gray area). An increase in the expression of some aminoacyl-tRNA ligases has been previously reported at the mRNA level in response to stress, and this effect has been causally linked to the UPR transcriptional effector ATF4 (Harding et al., 2003). This has been suggested to favor transcriptional recovery through increased tRNA charging (Krokowski et al., 2013). Our data support these observations and enlarge the pool of evidence available at the protein level (Bull and Thiede, 2012) by quantifying all cytosolic tRNA ligases and demonstrating that the vast majority of them show a large increase in expression in response to ER stress (Fig. 5B). Our dataset thus provides a direct measurement of the coordinate changes in expression of large complexes of interacting proteins during the UPR.

Although UPR activation is classically linked to translational attenuation through PERK-mediated phosphorylation of EIF2A, more recent data indicate that protein misfolding also causes transcriptional induction and an increase in protein synthesis (Han et al., 2013). We measured the rate of protein synthesis by means of the incorporation of puromycin into nascent polypeptides upon 18 h of tunicamycin treatment (Fig. S4A) (Schmidt et al., 2009). Under the conditions that we used to generate our resource database, protein synthesis is inhibited compared to control, thus making this an unlikely explanation for the increase in tRNA ligases in our model. Lower doses of tunicamycin did cause an increase in puromycin labelling, confirming the results described in other cellular models (Krokowski et al., 2013).

Our data show that key proteins involved in cell cycle regulation are clearly downregulated (Fig. 5A, green; see also Fig. 2). Among proteins significantly decreasing in expression we also find RNA polymerase II and a group of basic transcription factors and transcriptional regulators (light blue area). Among them are elongin B and C (TCEB2 and TCEB1, also known as ELOB and ELOC), two proteins forming the SIII complex, which interacts with RNA polymerase II (Pol II) and enhances its activity (Vos et al., 2018a). One exception in this group of interactors of Pol II, TCEA1 (also known as TFIIS) showed a large and significant dose-dependent increase (Fig. S4B, Table S8). We profiled the expression of general transcription factors and subunits of pre-initiation complexes that could be quantified in our dataset. Indeed, they were all downregulated upon tunicamycin treatment, with the exception of TCEA1, indicating that major rearrangements occur in the regulatory interactions of Pol II during ER stress (Vos et al., 2018b).

## DISCUSSION

We have generated a proteomic resource providing an accurate quantification of the UPR in HeLa cells comparing the effects of tunicamycin to those of other stressors inducing the UPR through different mechanisms, such as DTT and thapsigargin (Bergmann et al., 2018). While an activation of the UPR occurred upon treatment with all stressors used, only the SERCA inhibitor thapsigargin specifically perturbed cellular lipid metabolism. Interestingly, lipid biosynthesis is one of the specialized functions of the ER controlled by the UPR, which induces ER biogenesis. Thapsigargin could thus be used to selectively pinpoint the effects of the UPR on ER expansion, as well as on the recently described control of mitochondria-associated ER membranes (MAMs) and thus mitochondrial function (Saito and Imaizumi, 2018b).

Tunicamycin had the strongest effect of all stressors on the induction of UPR protein expression (Fig. 2). We show 133 proteins significantly upregulated by treatment with tunicamycin (5 µg/ml), including 15 with functional features similar to many known UPR targets (i.e. involved in ER import, folding, quality control and degradation, trafficking/Golgi), which had not been directly associated to the UPR to date. Together with the upregulation of proteins annotated as pro-apoptotic, consistent with the observation that prolonged exposure to tunicamycin causes programmed cell death (Zinszner et al., 1998), we measure the upregulation of several proteins of the secretory pathway and of the plasma membrane. These proteins may represent stalled cargo proteins, whose apparent expression increases as a result of inadequate secretion. Indeed, a number of membrane receptors with important roles in cell–cell and cell–matrix communication are also upregulated, possibly as consequence of defective sorting. Among them is discoidin domain receptor 1 (DDR1), a transmembrane tyrosine kinase acting as a receptor for collagen, and the EPHA receptor, which plays a key role in cell–cell communication. We anticipate that defective expression and localization of a number of these proteins might be causally linked to cell pathology in different contexts.

We have also detected a significant downregulation of many proteins in response to tunicamycin treatment and analyzed a potential role of RIDD in regulating this phenomenon. IRE1 can cleave mRNAs other than XBP1 during the UPR, which is suggested to reduce protein load on the ER (Hollien et al., 2009; Hollien and Weissman, 2006) and may contribute to apoptosis under irremediable ER stress (Han et al., 2009). Here, we were able to look at RIDD targets identified previously only in microarray experiments, and to assess whether their protein levels were also reduced. Taken together, our data confirm a poor correlation between protein and mRNA levels of previously identified RIDD targets.

Another novel aspect of the UPR highlighted in this dataset is the coordinated upregulation of many tRNA ligases. The molecular mechanisms underlying this increase might be linked to the finding that protein misfolding also causes transcriptional induction and an increase in protein synthesis. (Han and Kaufman, 2017). Using the incorporation of puromycin as a proxy to detect protein synthesis, we have not observed an increase in protein synthesis at the tunicamycin concentrations used to generate our proteomic database (Fig. S4A). We thus hypothesize that, in our system, the activation of autophagy, which is apparent in our data [Fig. 5, increase in SQSTM (SQSTM1, p62) and LAMP1; Table S8], generates an increase in the pool of free amino acids, which is matched by an increase in their loading process. It remains to be determined to what extent an inadequate supply of charged tRNA, e.g. due to a genetic defect, could be toxic under proteostatic stress.

In line with our data on protein synthesis, we have also observed that RNA Pol II and a group of basic transcription factors and transcriptional regulators significantly decrease in expression upon tunicamycin treatment. Among them are elongin B and C, two enhancers of RNA Pol II. Conversely, we observe that another interactor of RNA Pol II, TCEA1 has a large and significant increase in expression in response to tunicamycin. TCEA1 causes stalled RNA Pol II to overcome transcriptional blocks on template DNA by stimulating its endonuclease activity. This task is performed in cooperation with the Ccr4–Not complex, a global regulator of RNA Pol II transcription (Dutta et al., 2015). It could be envisaged that TCEA1 is selectively enriched at transcription sites during ER stress, possibly engaging in regulatory protein complexes with a different stoichiometry.

Taken together, measuring the expression of over 6200 proteins we (i) show common and specific proteomic features of UPR activation by tunicamycin, thapsigargin and DTT; (ii) define a subset of proteins that are upregulated with high significance in response to different doses of tunicamycin, some of which had not previously been linked to ER stress and (iii) highlight a coordinated upregulation of many tRNA ligases and stress-dependent changes in the regulatory complexes of RNA Pol II. These features could be both cause and effects of the cell pathology. As HeLa cells are extensively used as a model in cell biology and signaling, in-depth knowledge of the proteomic features of ER stress will provide support for the evaluation of results and experimental conditions, as well as to elucidate as-yet-unidentified outcomes of cell stress.

## MATERIALS AND METHODS

### Cell culture and SILAC labeling

HeLa cells from the American Tissue Culture Collection, recently authenticated and tested for contamination, were cultured in Dulbecco's modified Eagle's medium, supplemented with 4500 mg/l glucose, 110 mg/l sodium pyruvate, 600 mg/l L-glutamine and 10% FBS (Life Technologies-GIBCO). For SILAC labeling, cells were cultured in DMEM with the same formula but lacking the two amino acids lysine and arginine (SILAC medium, GE Healthcare) and with dialysed FBS in order to avoid amino acid carry-over. The medium was supplemented with either ‘light’ unlabeled or ‘heavy’ isotope-labeled ^13^C_6_^15^N_2_–lysine (Lys8) and ^13^C_6_^15^N_4_–arginine (Arg10) (Cambridge Isotope Laboratories). Cells were cultured for more than 10 passages in heavy medium and almost complete incorporation of heavy amino acids in the cells was confirmed by MS analysis. Labeled cells were aliquoted and frozen. The experiments for the three biological replicates were performed using different cell aliquots at different passages over a time period of months. Tunicamycin and DTT were purchased from Sigma-Aldrich, thapsigargin from SERVA Electrophoresis. Tunicamycin concentrations were as follows: 0.125, 0.25, 0.625, 1.25, 2.5 or 5 µg/ml. The highest concentration used, 5 µg/ml corresponds to ∼6.12 µM, considering that tunicamycin contains four main homologous antibiotics of varying molecular weight (817–859 Da).

### Cell lysis and spike-in SILAC mix

HeLa cells were lysed in a buffer consisting of 0.1 M Tris-HCl pH 8.0, 0.1 M DTT and 4% SDS at 95°C for 5 min. After chilling to room temperature, the lysates were sonicated using a Branson-type sonicator and then clarified by centrifugation at 16,000 ***g*** for 10 min. Protein content was determined by comparison to a tryptophan protein standard using a spectrophotometric method, with excitation wavelength 280 nm and emission wavelength 350 nm. A heavy SILAC standard was prepared by mixing heavy HeLa cells under a variety of conditions in order to cover the proteome of stressed and unstressed cells. Specifically, we mixed equal amounts of lysates from untreated and stressor-treated heavy HeLa cells to obtain a master mix. For each sample, 100 μg of light cell lysate was mixed with 100 μg of heavy master mix and further processed.

### Protein digestion

Proteins were digested using the filter aided sample prep (FASP) method (Wisniewski et al., 2009). Briefly, cell monolayers were lysed in 4% (w/v) SDS, 100 mM Tris-HCl pH 7.6, 0.1 M DTT. 200 μg of protein was loaded onto Microcon YM-30 cartridges (Millipore). SDS was replaced by washing 2–3 times with buffer containing 8 M urea (Sigma-Aldrich) in 0.1 M Tris-HCl pH 8.5. The proteins were subsequently alkylated by adding 0.05 M iodoacetamide to the urea buffer, and the excess reagent was removed by filtration. The reduced and alkylated proteins were digested using trypsin (Promega) with an enzyme-to-protein ratio of 1:100. Trypsin generates peptides of average length 7–20 amino acids with a strong C-terminal charge, ideally suited for MS analysis. Peptides obtained by FASP were eluted from the filter with 0.05 M NH_4_HCO_3_ in water and desalted using a C18 membrane (Thermo Fisher Scientific) and stop and go extraction (stage) tips (home made).

### MS data acquisition and analysis

Eluted peptides (3 μg/sample) were separated on a reverse phase 50-cm column with 75 μm inner diameter, packed in-house with 1.8 μm C18 particles (Dr Maisch GmbH) kept at 50°C by a column oven (Sonation). Liquid chromatography was performed on an EASY-nLC 1000 ultra-high pressure system was coupled through a nanoelectrospray source to a Q Exactive mass spectrometer, applying a nonlinear 270 min gradient of 2–60% buffer B [0.1% (v/v) formic acid, 80% (v/v) acetonitrile] at a flow rate of 250 nl/min (all Thermo Fisher Scientific). Data were acquired in data-dependent mode. The survey scans were acquired at a resolution of 70,000 at m/z=200 in the Orbitrap analyzer. The top 10 most abundant isotope patterns with charge ≥2 from the survey scan were selected with an isolation window of 1.6 Thomson and fragmented by higher energy collisional dissociation (Top 10). The maximum ion injection times for the survey scan and the MS/MS scans were 20 and 60 ms, respectively, and the ion target value for both scan modes were set to 3E6 and 1E6, respectively. Repeated sequencing of peptides was kept to a minimum by dynamic exclusion of the sequenced peptides for 45 s. The dataset comparing different stressors was obtained using a Q Exactive HF instrument after separation by means of a linear gradient of buffer B over 120 min, using a Top 15 method with an injection time of 20 ms for survey scans and 25 ms for MS/MS scans.

### Computational proteomics and data analysis

MaxQuant software (version 1.5.3.2) was used for the analysis of raw files (Cox and Mann, 2008). Peak lists were searched against the human UniProt FASTA database version of 2012 (88,976 entries) and a common contaminants database (247 entries) using the Andromeda search engine (Cox et al., 2011). False discovery rate was set to 1% for peptides (minimum length of 7 amino acids) and proteins, and was determined by searching a reverse database. A maximum of two missed cleavages were allowed in the database search. Peptide identification was performed with an allowed initial precursor mass deviation up to 7 ppm and an allowed fragment mass deviation of 20 ppm. The ‘Match between runs’ option in MaxQuant was activated. The shotgun proteomics approach is based on the measurement of the spectra of individual peptides, which are then assembled into proteins. MaxQuant employs a ‘target-decoy search strategy’ to control for false-positive peptide identifications and the concept of ‘posterior error probability’ (PEP) to control the quality of a peptide spectrum. The PEP score integrates individual peptide properties, such as length and charge, with the score provided by the Andromeda search engine. Further *in silico* verification of the proteomic data included several parameters of quality control for all data points in the dataset: (i) number of valid SILAC ratios in each sample (3394±791, mean±s.d.); verification of consistency and reproducibility for all samples by calculating (ii) the median SILAC ratio of each sample (1.006±0.002) and (iii) the sum of SILAC ratios of each sample (3526±823). The number of quantified peptides was 48744±1768 for the light lysates and 48600±1761 for the heavy lysates.

The mass spectrometry proteomics data have been deposited to the ProteomeXchange Consortium via the PRIDE partner repository with the dataset identifier PXD013496 (http://www.ebi.ac.uk/pride/archive/projects/PXD013496).

### Statistical analysis

Data analysis was performed with the Perseus software (version 1.5.1.2) embedded in the MaxQuant environment (Tyanova et al., 2016). SILAC ratios were used without further normalization. Where indicated, the data were logarithmized (Log_2_ SILAC ratio). Categorical annotations were supplied in the form of UniProt Keywords, KEGG and Gene Ontology. SILAC ratios (light:heavy) were used for data analysis. The list of proteins significantly changing upon tunicamycin treatment was obtained using a paired *t*-test between vehicle treated and tunicamycin (5 μg/ml)-treated samples, using permutation-based FDR at 0.05, performing 250 randomizations; grouping of technical replicates was preserved in randomization; the *s*_0_ parameter was set to 0.1, a condition ensuring that the UPR protein calnexin is among the significant hits. Data were filtered for 75% valid values before test. Technical replicate two of biological replicate two was excluded from the statistical analysis due to overall lower signal intensity (see Fig. S2F). Hierarchical clustering was performed on significant proteins, using Fisher's exact test to calculate enrichments in categorical terms. Enrichments in the network were calculated by Fisher's exact test using Benjamini-Hochberg false discovery rate for truncation, setting a value of 0.02 as threshold.

### Interaction network analysis

Interaction network analysis of the tunicamycin significantly regulated proteins was performed by taking advantage of the protein–protein interaction database STRING (version 10.0). We have used medium to high confidence (0.5–0.7) and filtered interactions keeping only the ones derived from experiments (known interactions experimentally determined). The resulting network was visualized by using Cytoscape 3.2.1 where we then overlaid the tunicamycin-induced changes of protein expression.

### Western blotting

HeLa cell lysates, 20 μg/sample, were run on a 4–12% Bis-Tris polyacrylamide gels using MES as a trailing ion in the buffer (Invitrogen). Proteins were transferred to a nitrocellulose membrane (Whatman) using Towbin buffer with 20% (v/v) methanol in a semi-dry transfer apparatus (Hoefer) and reversibly stained with Ponceau S for loading control. Membranes were incubated in TBS buffer containing 0.01% Tween-20 and 5% (w/v) skim milk powder (Fluka), containing the primary antibodies. The following antibodies were used: ER stress antibody sampler kit (all 1:1000; #9956, Cell Signaling Technology; including secondary antibodies used here), anti-MAFF (1:500; AV38984, Sigma-Aldrich), anti-eIF4G1 (1:1000; 2858S, Cell Signaling Technology), anti-tubulin (1:1000; sc-9104, Santa Cruz Biotechnology). Puromycin incorporation was detected using the 12D5 mouse monoclonal antibody (MABE343, Millipore) at a 1:5000 final concentration. The quantification of gel bands was performed using the image processing program ImageJ (http://imagej.nih.gov/ij) (Schneider et al., 2012).

## Supplementary Material

Supplementary information
