# Supplementary material for: SILAC-based quantitative proteomics using mass spectrometry quantifies endoplasmic reticulum stress in whole HeLa cells
Source: Dis Model Mech. 2019 Nov 11;12(11):dmm040741. doi: 10.1242/dmm.040741 (PMC6899043; doi:10.1242/dmm.040741)
Supplement: Supplementary information [file dmm-12-040741-s1.pdf]

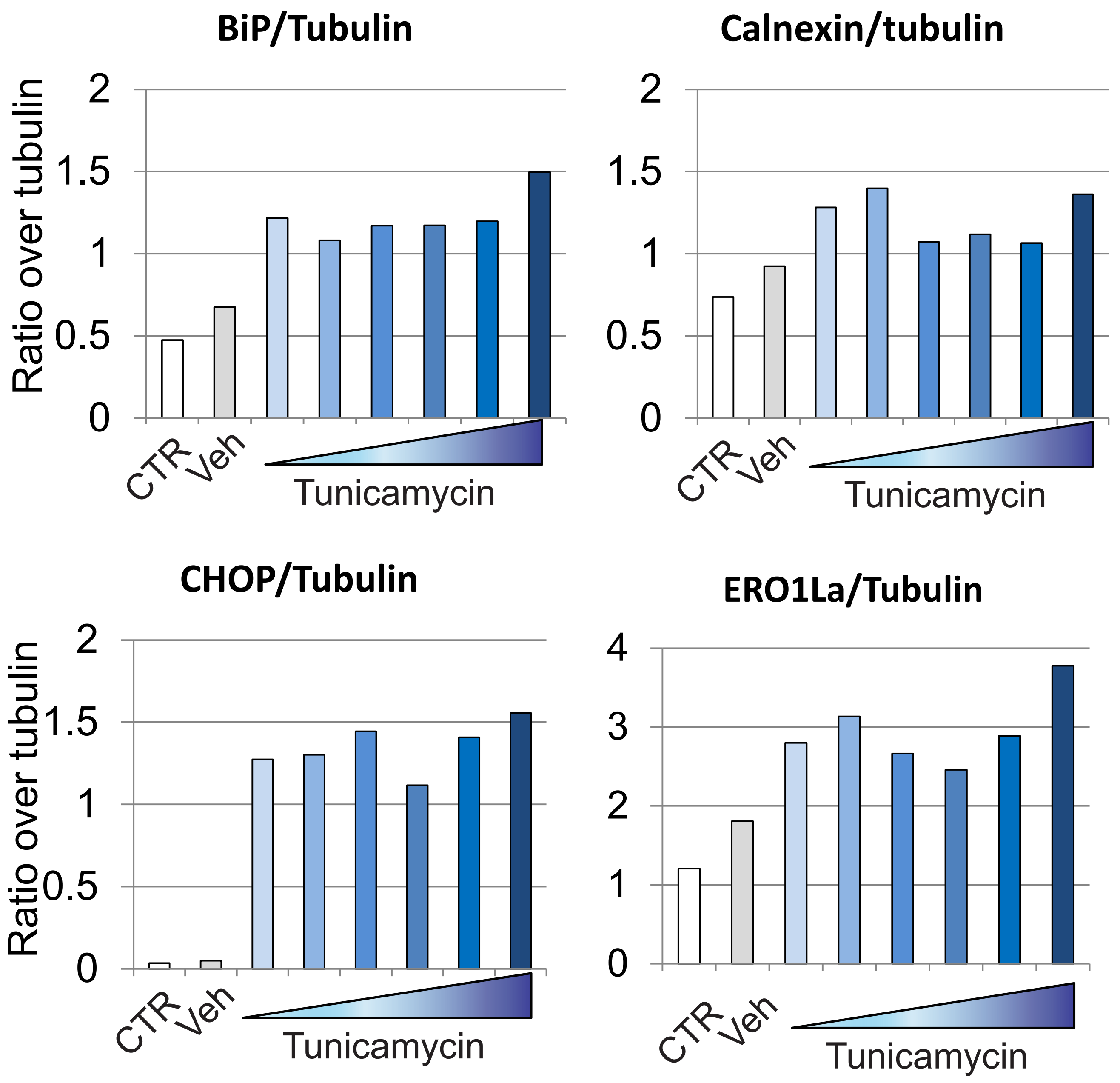


Figure S1. Quantification of total levels of selected UPR proteins relative to tubulin (as loading control). The densitometry analysis was carried out on the western blots shown in Fig. 1 using ImageJ.

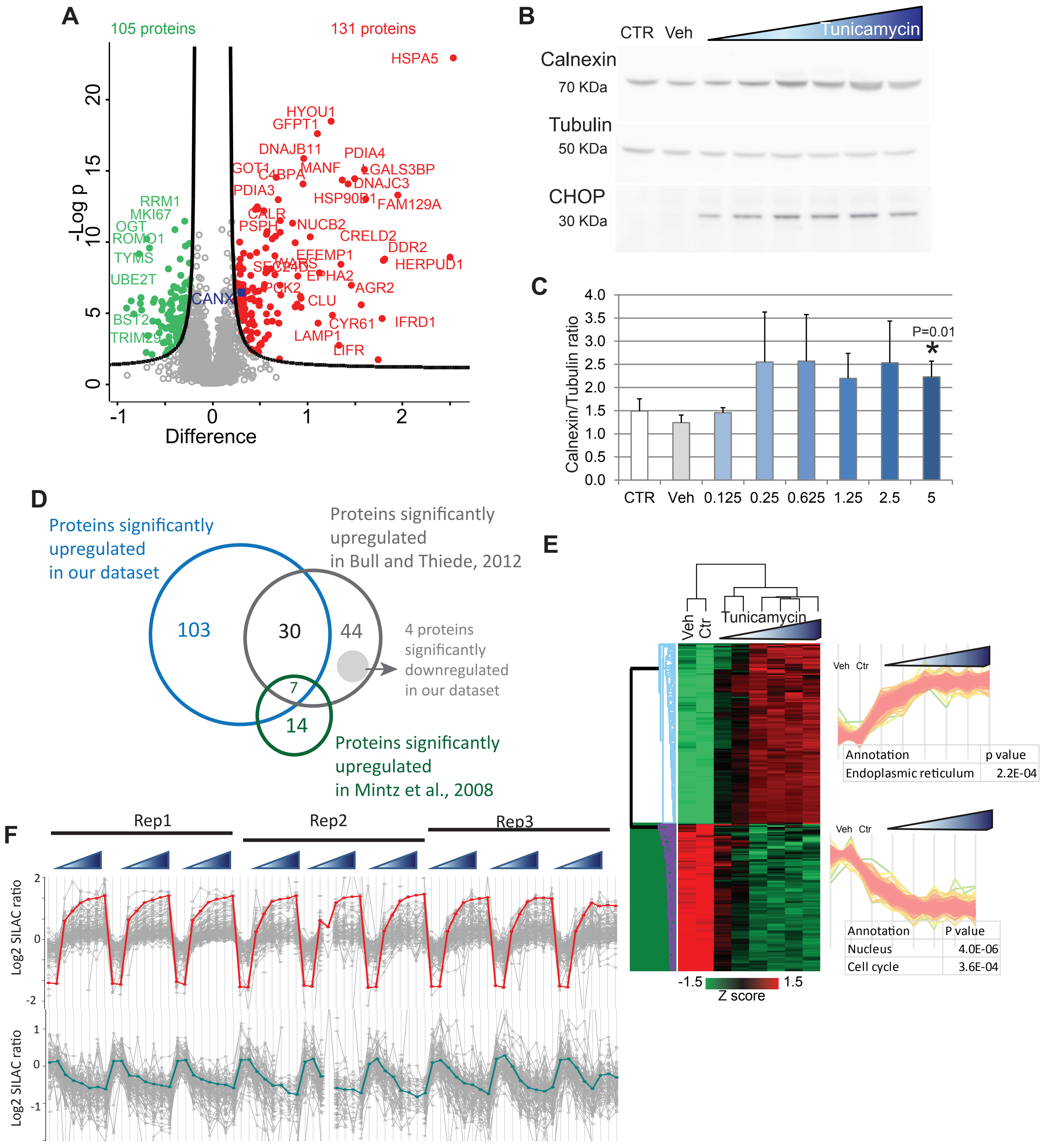


Figure S2. (A) Volcano plot comparing samples treated with vehicle to those with 0.625 µg/ml tunicamycin. Proteins significantly upregulated upon tunicamycin treatment are shown as red dots, those downregulated as green dots. The curve is derived at FDR=0.05 and S0=0.5 as described in the Methods section. (B) Western blot analysis of the expression of Calnexin in HeLa cells treated with increasing concentrations of Tunicamycin. The same lysates used for quantitative proteomics were analysed. The expression of CHOP/GADD153, a known component of the ER stress-mediated apoptosis pathway, is shown in parallel. (C) Quantification of the increase in the expression of Calnexin. The average grey intensity of each calnexin band was quantified in a pre-set area of fixed size and normalized to the corresponding intensity of the tubulin bands, used as a loading control (see panel A). Data are represented as mean +/- SD of three western blots from three biological replicates. (*) significantly different from Vehicle based on Student's T-Test. (D) Number of proteins significantly upregulated upon tunicamycin treatment in our dataset (blue) compared to previous reports with a similar experimental approach (grey and green). Partial lack of correspondence is due to proteins that resulted downregulated in our dataset (solid grey circle). (E) Unsupervised hierarchical clustering of proteins significantly different between DMSO and tunicamycin (0.625 µg/ml)-treated HeLa cells (median of N=3 biological replicates). (F) Expression profiles of significantly regulated proteins from the lists in Supplementary tables S5 and S6. All three biological replicates, each with three technical replicates respectively are shown. The evident drop in intensity and proteins detection in part of technical replicate 2 of biological replicate 2 led to removal of these data points from the statistical analysis used to generate table S3.

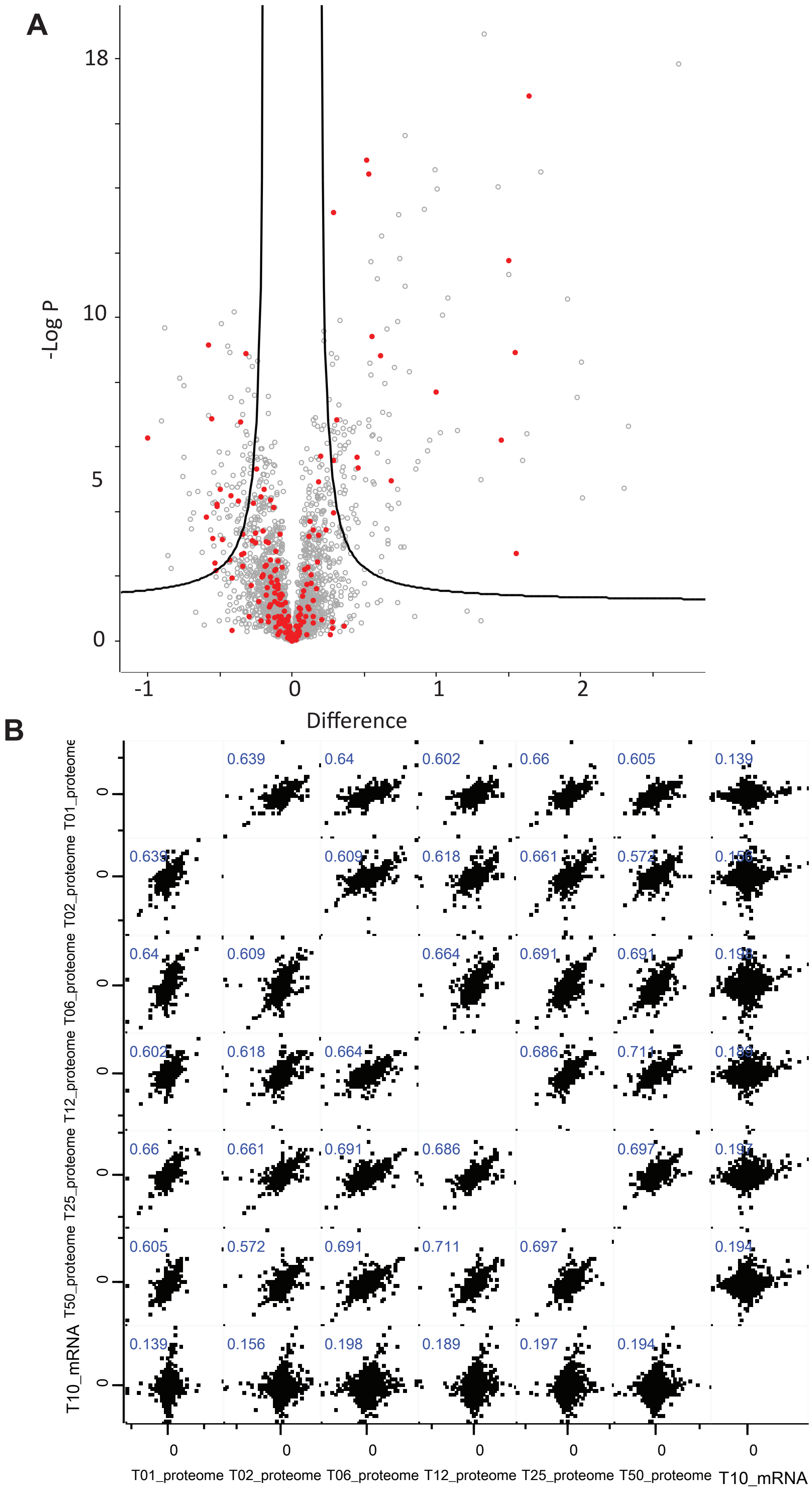


Figure S3. (A) Volcano plot showing the distribution of RIDD targets (red dots). P values (-log10) are plotted versus difference (log2). (B) Correlation between our proteomics dataset and the mRNA data of Saito et al (Ref. 31). For each replicate we compare transcripts and proteins. The analysis revealed a correlation of about 0.68 for proteomes and of about 0.16 on average between proteins and mRNAs

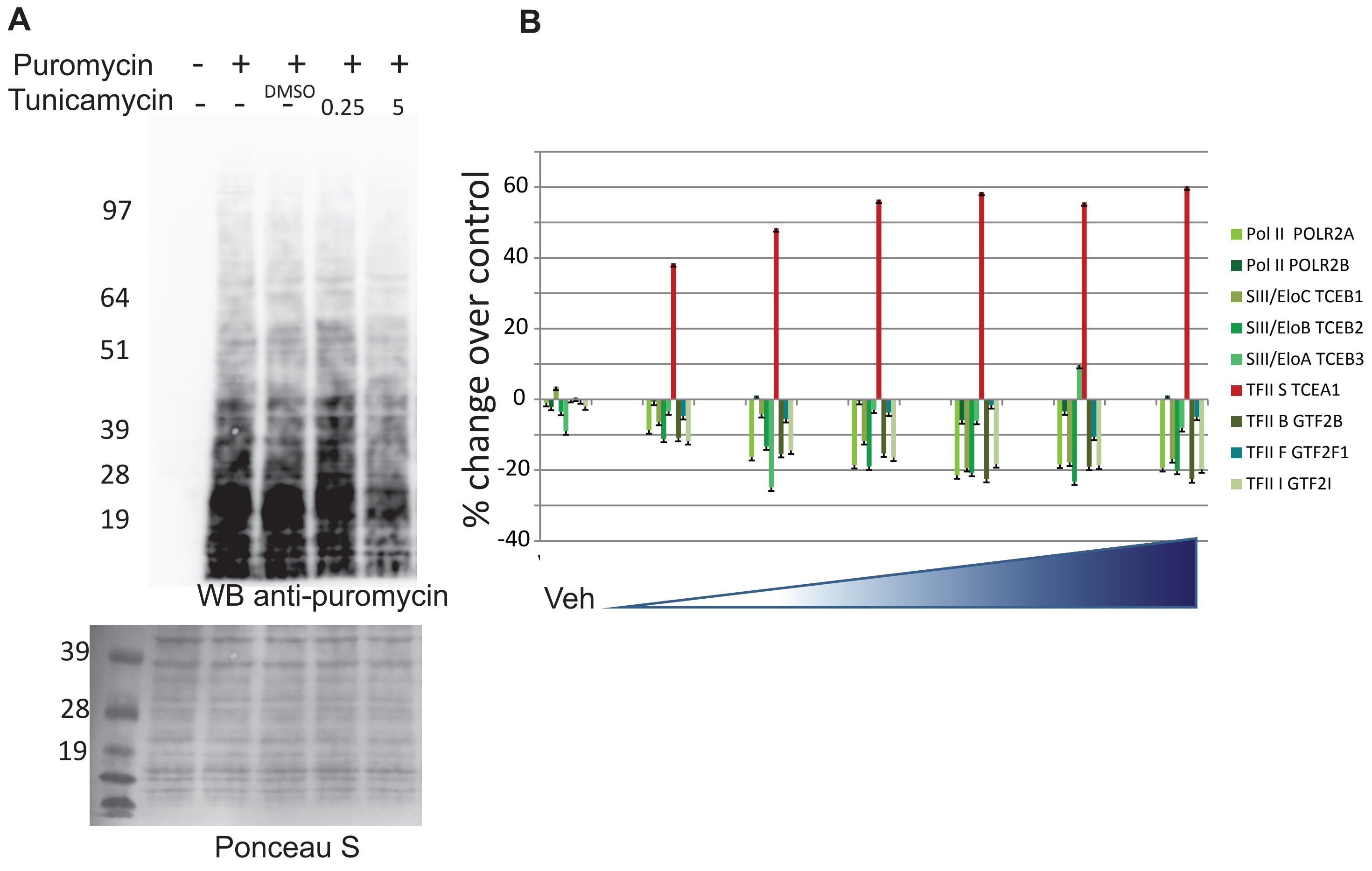


Figure S4. (A) Inhibition of protein synthesis at high tunicamycin concentrations. Cells were treated as indicated for 18 hs. Puromycin (10 ug/ml) was added to the culture medium for the last 30 minutes of incubation. Cells were lysed and processed for immunoblot analysis using an antibody specific for puromycin (see Schmidt et al., 2009 in the main text). Ponceau-S staining indicates equal loading of each lane. (B) Percent variations were derived for vehicle and each tunicamycin concentration with respect to the untreated control sample. Data are the average +/-SD of three biological replicates.

Disease Models & Mechanisms • Supplementary information

**Table S1.** Complete dataset with all biological and technical replicates. Expression values are $log_2$ SILAC ratios (light/heavy, see figure 1). To import the table into the Perseus program (free download at http://www.perseus-framework.org) convert to txt format. Expression data columns should be imported as “main”, annotations as “categorical”, peptide numbers and intensity as “numerical”. Text columns (gene names, protein names) will be automatically recognized.

Click here to Download Table S1

**Table S2**. List of all significantly enriched terms form the 2D annotation enrichment shown in figure 1c. To import the table into the Perseus program, convert to txt format. See description in Table S1.

Click here to Download Table S2

**Table S3.** Complete dataset averaged by biological replicate (SILAC ratios). Instructions for direct import into Perseus and the use of the profile plots option are described in the legend of Table S1.

Click here to Download Table S3

**Table S4**. Complete list of annotation enrichments resulting from the cluster analysis in Figure 2D.

Click here to Download Table S4

**Table S5**. 100 profiles most similar to HSPA5/Bip by correlation.

Click here to Download Table S5

**Table S6**. 100 profiles most similar to MKI67 by correlation.

Click here to Download Table S6

**Table S7**. Complete list of annotation enrichments resulting from the cluster analysis in Figure 3. Proteins are listed in two groups according to the test difference (up- and downregulated) as indicated.

Click here to Download Table S7

**Table S8.** Significantly upregulated proteins with complete annotations (description in text).

Click here to Download Table S8

**Table S9.** Significantly downregulated proteins with complete annotations (description in text).

Click here to Download Table S9

Disease Models & Mechanisms • Supplementary information
